# Prevalence and associated factors of caesarian section in Ethiopia: a multilevel analysis of the 2019 Ethiopia Mini Demographic Health Survey

**DOI:** 10.1186/s12884-021-04266-7

**Published:** 2021-11-30

**Authors:** Samuel Hailegebreal, Girma Gilano, Binyam Tariku Seboka, Mohammedjud Hassen Ahmed, Atsedu Endale Simegn, Getanew Aschalew Tesfa, Delelegn Emwodew Yehualashet

**Affiliations:** 1 Department of Health Informatics, College of Medicine and Health Sciences, School of Public Health, Arba Minch University, Arba Minch, Ethiopia; 2 Department of Health Informatics, College of Medicine and Health Sciences, Dilla University, Dilla, Ethiopia; 3 Department of Health Informatics, Institute of Public Health, Mettu University, Mettu, Ethiopia; 4 Department of Anesthesia, College of Medicine and Health Science, Wachemo University, Hossana, Ethiopia

**Keywords:** Caesarian section, Prevalence, Multilevel, Ethiopia

## Abstract

**Background:**

Caesarian section is a vital emergency obstetric intervention for saving the lives of mothers and newborns. However, factors which are responsible for caesarian section (CS) were not well established in the country level data. Therefore, this study aimed to assess the prevalence and associated factors of caesarian section in Ethiopia.

**Methods:**

Data from the Ethiopian Mini Demographic and Health survey 2019 were used to identify factors associated with the caesarian section in Ethiopia. We applied multi-level logistic regression and a *p*-value of <0.25 to include variables before modeling and a *p*-value<0.05 with 95% confidence interval (CI) for final results.

**Result:**

The prevalence of caesarian section in Ethiopia was 5.44% (95% CI; 0.048-0.06) in2019. Women in age group of 30-39 and 40-49 years had a higher odd of caesarian section (AOR = 2.14, 95%CI = 1.55-2.94) and (AOR = 2, 95%CI = 1.20-3.97) respectively compared to women in age group of 15-29 years. Women with secondary and higher educational level had higher odds of caesarian section (AOR = 2.15, 95%CI = 1.38-3.34) and (AOR = 2.8, 95%CI = 1.73-4.53) compared to those in no education category. Compared to Orthodox, Muslims and Protestant religions had lower odds of caesarian section with AOR of 0.50 (0.34-0.73) and 0.53 (0.34-0.85). Having <2 births was also associated with the low caesarian section 0.61(0.52-1.22). Using modern contraceptive methods, having ANC visits of 1-3, 4^th^, 5 plus, and urban residence were associated with higher odds of caesarian section as 1.4 (1.05-1.80]), 2.2 (1.51-3.12), 1.7 (1.12-2.46), and 2.4 (1.65-3.44) 1.6(1.04-2.57) respectively.

**Conclusion:**

Although evidence indicates that the caesarian deliveries increased both in developed and underdeveloped countries, the current magnitude of this service was very low in Ethiopia which might indicate missing opportunities that might costing lives of mothers and newborns. Women’s age, religion, educational status, parity, contraceptive method, and ANC visit were individual level factors influenced caesarian section. whereas, region and place of residence were community level factors affected caesarian section in the country. Depending on these factors, the country needs policy decisions for further national level interventions.

**Supplementary Information:**

The online version contains supplementary material available at 10.1186/s12884-021-04266-7.

## Introduction

Caesarian section (CS) is the delivery of the pregnancy outcome by incision through the maternal abdomen and uterus which was named after the belief that Julius Caesar was born through CS. It has been recommended for the mothers whose labor progress is poor due to the either maternal or fetal factors [1]. Report on the CS rate from 1990 to 2018 for countries all over the world indicated that rate of CS continues to rise in high, middle, and low-income countries [2]. Evidence from Saudi and India also indicated that there was a rise in the CS deliveries due to various reasons [3, 4]. In Sub-Saharan African countries, there was no consistent increase throughout the region; however, there CS was still rising [5]. In Ethiopia, the small size studies indicates there was also a rise in performing CS in the country [6, 7].

According to the World Health Organization (WHO) caesarian section statement, the optimal proportion of caesarian section was considered to range between 10-15%. Since 1985, caesarian section has increased in both developing and developed countries. The maternal and newborn deaths were significantly decreased until the proportion become 10% [8]; however, evidence indicated that the rate above 10% was not linked to any further improvement in the population based assessments [9]. Caesarian section should be only performed when medically necessary for the patient care as it also poses some complication like disability and death. Both under and over performed caesarian section have adverse outcomes on the health of the mothers and newborns [10]. Countries need to control the over performance of caesarian section but also make sure that life is not losing while these services are available. Under performance costs unnecessary lives of mothers and newborns, while over performance exposes mothers and newborns to extra unwanted complications [8, 10, 11]. To ensure cautious use of this service, prevalence and reason of indication (factors) might be vital gates in developing countries to regulate the event.

Overall, the caesarian section in Ethiopia were 21% in Butajira General hospital in 2019 [12]; 29.7% in Jugal hospital in 2019 [6]; 47.6% in Dessie town hospitals [4]; 34.3% in hospitals in Harar [13]; 30.9% public hospitals in Northern Ethiopia [14]; 24.7% in Durame general hospital [15]; 28.1% in three hospitals of Southwest Ethiopia [16]; 27.6% in Atta hospital [17, 18]; 38.5% in a national cross-sectional study [19]; 3.5% in another community-based cross-sectional study [20]; 51.9% in South Gondar [21]. The rate of the caesarian section was 18% in 2011. It was higher in private and profit institutions, while maternal indication was also higher than all other indications in both private and public hospitals [22]. A study conducted in the Western Wellega (Ethiopia) showed that the prevalence of the cesarean section was 33.1%, where maternal age, gestational age, and fetal weight essentially prejudiced magnitude [7]. Some evidence showed that the caesarian section has been carried out in favor of women economic status [23]. For instance, Addis Ababa (the capital) had the highest caesarian section proportion [24]

There are variety of factors associated with CS in the country. Women's education, wealth index, parity, delivery were all responsible for higher caesarian section [25]. The prevalence of caesarian section in Bahirdar, Northern Ethiopia was 41.8%, and factors like delivery in private health facilities, breech presentation, urban residence, being referred, being government employees, and parity associated with the increased magnitude [26]. The caesarian section was also performed more among obese women as revealed by one study [27]. In Addis Ababa, the prevalence of caesarian section was 38.3%, while having some risk factors like delivering in private health facility and maternal education were associated in the rural areas [28].

The proportion was also very high (49.3%) in Hawassa city (the capital of Southern Ethiopia), where monthly income above poverty, and previous pregnancy complications were positively associated unlike the use of partograph which lowered the proportion [29]. In another study, singleton pregnancy, birth weight less than 2500gm, completely documented partograph, and pregnancy-induced hypertension were found to be the determinants of caesarian section [30].

The meta-analysis of studies done in the country presented the pooled prevalence of 29.55% Caesarean sections with the cephalopelvic disproportion and non-reassuring fetal heart rate pattern were associate [31]. Institutional deliveries were accompanied by a caesarian section (24.8 %.) in other study [32].

Although factors associated with the caesarian section look identified, most studies in the process were used traditional single level analyses which were at risk of higher bias compared to the multilevel analyses. Most studies were also followed analysis of the pocket places or residences and had no regional comparison which made them very limited in that context and also put factors associated with caesarian section in cloud.

Using the country representative sample have the paramount advantages over other small area studies in determining the compiled magnitude to display national level factors and might led to the further policy decisions for national level interventions. Therefore, this study aimed to assess the prevalence and associated factors of caesarian section in Ethiopia.

## Methods

### Study design, setting, and period

A cross-sectional study was conducted using Ethiopia Mini Demographic Health Survey (EMDHS) 2019. Ethiopia is the second most populous country in African continent situated at (3^o^-14^o^N, 33^o^ – 48°E). EMDHS is the country representative sample survey carried out between EDHS. The country has nine regions and two city administrations which further categorized in to contextual groups as agrarian (Amhara, Benishangul-Gumuz, Gambela, Harari, Oromia, Southern Nations, Nationalities, and People Region (SNNPR), and Tigray), pastoralists (Afar and Somali), and city administrations (Addis Ababa and Dire-Dawa). We downloaded a secondary data from DHS website (www.dhsprogram.com). The detailed sampling procedure has been presented in the full EMDHS 2019 report [33].

### Study variables

The outcome variable was caesarean section delivery. It was retrieved from dichotomized DHS question asking for ‘delivery by caesarian section’. The responses were classified originally and here as No (0) or yes (1).

### Independent variables

Independent predictors were classified individual and community-level factors. Individual level factors were a religion, contraception, age, maternal education, household wealth index, place of delivery, parity, birth order, preceding birth interval, number of ANC visit and sex of the child. Community level factors were region and residence. The detailed information about explanatory factors were presented in Table 1.

**Table 1:** The summary of some of the independent variables

Variables	Definition/categories	Reference
Age of the mother	The of the mother was coded 15-19, 20-24, 25-29, 30-34, 35-39, 40-44, and 45-49; however, during cleaning some of the categories lack participants or very limited, we recoded to 15-29, 30-39, 40-49	15-19
Mother educational status	No-education, Primary, Secondary, and Higher	No-education
Religion	Religion was Protestant, Orthodox, Muslim, Catholic, tradition, other. Depending on the number of participants we merged Catholic, Traditional, and Other and the new coding become Protestant, Orthodox, Muslim and others	Orthodox
Marital status	Marital status was coded as never in union, married, living with partner, widowed, and divorced, no longer living together/separated; however, number of participants from the last four was very few, thus, we categorized unmarried and married by living married alone and classified all else.	Unmarried
Place of delivery	It was coded as respondent’s home, other home, government hospital, government health center, government health post, other public health sector, private hospital, private clinic, Ngo health facility, NGO other health facility, and other. Because of statistically insignificant number of participant and some other similarities, we recoded to home and institutional delivery.	Home
House wealth index	In EMDHS, household wealth index was categorized in quintiles as: poorest, poor, average, rich and richest and for this category, they used principal component analysis to make this category. Then, for easy decision making we re-categorized the scale in to poor, middle, and rich	Poor
Birth order	It a count number ranging from 1 to 15; however, we categorized in to first, 2^nd^, and >=3	First
Parity	no birth, one birth, two birth, and three and above births	No birth
Number of ANC	ANC coded as count number ranging from 1 to 20; however, to assist good decision making we recoded into No-visit, 1-3 visit, 4th –visit, and 5+- visit	No visit
preceding birth interval	Preceding birth interval was coded from 1 to 219 months in the dataset. We recoded in to <15, 15-30,>30 months for the same reason as above	<15
Contraception	EMDHS coded the variable No-method, Traditional method, Modern method	No method

### Data management and Statistical analysis

Before conducting the descriptive data analysis, we weighted the data to adjust for the non-proportional allocation of samples to strata and regions. Then, descriptive statistics were performed and presented using weighted frequencies, mean ± (standard deviations), and percentage, while all analyses were performed using STATA version 15 (STATA Corporation. IC., TX, USA).

All the fixed effect factors were introduced in to model after bivariate test at *p*-value of <0.25; all random effect variables were also tested similarly. In the final model, the association of the predictors with caesarian section was checked by considering *p*-value of <0.05 and the results were presented using AOR with 95% CI.

To identify community effect, Intra-cluster Correlation (ICC) was valued by the community level variance. Then the Likelihood Ratio (LR) test, Median Odds Ratio (MOR), and Proportional Change in Variance (PCV) were examined for the fitness of the model as follow.

ICC= $\frac{{\upsigma^2}_a}{{\upsigma^2}_a+{\upsigma^2}_b}$; where, σ^2^_*a*_ (random effect) is the community level variance and σ^2^_*b*_ (fixed effect) indicates individual level variance and ( σ^2^_*b*_ ) equal to π^2^/3.

Median Odds Ratio (MOR) was estimated as MOR= *e* ^0.95*^ $\sqrt{V{a}_{\_1}}$, where, *Va*__1_ is community variance, MOR used to measure an unexplained cluster heterogeneity. Proportional Change in Variance (PCV) was estimated as PVC = $\frac{Va\_1- Va\_2}{Va\_1}$ , PCV measures the total variation attributed by individual-level factors and community-level factors.

Since four consecutive models were built to fit the data, the model comparison was done using the Likelihood Ratio (LR) test, while goodness of fit was assessed using deviance (-2LL).

## Result

### Sociodemographic characteristics of study participant

A total of 5527 study participant were involved in this study. Out of this 300 (5.44%) were underwent caesarian section in the country in 2019. Among the eligible women for caesarian section 38 % were Muslim religion follower. More than half of the study participants were in age group of 15-29 (55.2 %) and had no education (53.58%). Majority of the women were from Oromia (40%), followed by SNNP (20%). Majority of the participants (95.77%)) were married and 45.56% of the women were poor (Table 2).

**Table 2:** Socio- demographic characteristics of study participant (Weighted, *N*=5,527)

Variables	Weighted frequency	Percent
**Region**		
Tigray	371	6.72
Afar	86	1.55
Amhara	1050	19
Oromia	2211	40
Somali	409	7.39
Benishangul	67	1.22
SNNPR	1106	20.01
Gambella	25	0.45
Harari	16	0.3
Addis Ababa	156	2.83
Diredawa	30	0.54
**Residence**		
Rural	1,367	24.73
Urban	4,160	75.27
**Age**		
15-29	3,050	55.19
30-39	2,000	36.19
40-49	476	8.62
**Religion**		
Orthodox	1860	33.65
Muslim	2100	38
Protestant	1460	26.42
Others	106	1.92
**Marital status**		
Unmarried	234	4.23
Married	5293	95.77
**Educational status**		
No-education	2962	53.58
Primary	1956	35.4
Secondary	415	7.51
Higher	194	3.51
**Wealth status**		
Poor	2,518	45.56
Middle	1,044	18.88
Rich	1,965	35.55

### Associated factors of caesarian section

#### The random effect analysis result

The ICC in the null model indicated that 41% of the total variability in caesarian section was attributed due to the differences between clusters while the remaining unexplained 59% of the total variability of caesarian section was attributed to the individual differences. Also, the MOR was 3.9 in null model which indicate that there was a variation in caesarian section between clusters. If we randomly select two women from different clusters, if we transfer women from low caesarian section clusters to higher caesarian section clusters, she might have 3.9 times higher odds of having caesarian section. This showed that the existence of significant heterogeneity in caesarian section delivery. The proportional change in variance (PCV) in this model was 77% which showed that 77% of community variance observed in the null model was explained by both community and individual level variables. The best fitted model was compared by deviance and the best fitted was model III, with the lowest deviance (Table 3).

**Table 3:** Random effect analysis result

Parameter	Null model	Model I	Model II	Model III
Community-level variance	2.31	0.71	0.90	0.52
Loglikelihood	-1202	- 1089.89	- 1139.18	- 1068.8
Deviance	2404	2179.78	2278.36	2137.6
MOR	3.9	2.2	2.4	1.9
PVC	Ref	69%	61%	77%
ICC	0.41	0.18	0.22	0.14

#### The fixed effect analysis result

In the multilevel logistic regression analysis, women age, religion, educational status, parity, contraceptive method, ANC visit, region and place of residence were significantly associated with caesarian section delivery. The odds of CS among women in age group 30-39 and 40-49 years were 2.14 (AOR = 2.14, 95%CI = 1.55-2.94) and 2.20(AOR = 2.20, 95%CI = 1.20-3.97) times higher than that of the women in age group of 15-29 respectively. The odds of having CS among Muslim women (AOR = 0.50, 95%CI = 0.34-0.73) and Protestant (AOR = 0.53, 95%CI = 0.34-0.85) religion followers were decreased by 50% and 47 % compared to the Orthodox religion followers respectively. The odds of CS among women who educated secondary and higher level were 2.15 (AOR = 2.15, 95%CI = 1.38-3.34) and 2.8 (AOR = 2.8, 95%CI = 1.73-4.53) times higher than that of women with no education.

The odds of CS among mothers with 3-5 parity (AOR = 0.61, 95%CI = 0.44- 0.94) was decreased by 39% than that of mothers with 1-2 parity.

Women who use modern contraceptive methods had higher odds of CS (AOR = 1.4, 95%CI = 1.05-1.80) than women who had no contraceptive use experience. Women who used antenatal care of 1-3 visit, four visits, and five and above visits had higher odds of CS with AOR of (AOR = 2.2, 95% CI = 1.51-3.12), (AOR = 1.7, 95% CI = 1.12-2.46) (AOR = 2.4, 95% CI = 1.65-3.44) than those who do not have ANC respectively.

The odds of the CS for the women residing in Addis Ababa (AOR = 3.4, 95% CI = 1.46-7.22), Amhara (AOR = 2.2, 95% CI = 1.07-4.45), Dire Dawa (AOR = 4.9, 95% CI = 2.26-10.56), Harari (AOR = 2.9, 95% CI = 1.55-2.94), and SNNP (AOR = 2.8, 95%CI = 1.26-6.00) were higher than that of the women residing in Tigray. The odds of having CS among women of the urban resident was 1.6 (AOR = 1.6, 95% CI = 0.41, 0.45) relative to the rural resident (Table 4).

**Table 4:** Factors associated with caesarian in Ethiopia, EMDHS 2019

Variables	Null model	Model 1	Model 2	Model 3
**Individual- level variables**				
**Women age**				
15-29	**-**	Ref	**-**	Ref
30-39	**-**	2.3 [1.66-3.14] **	**-**	2.14 [1.55-2.94] **
40-49		2.4 [1.32-4.34] **	**-**	2.2 [ 1.20-3.97] *
**Religion**				
Orthodox	**-**	Ref	**-**	Ref
Muslim	**-**	0.58 [0.41-0.81] **	**-**	0.50 [0.34-0.73] **
Protestant	**-**	0.55 [0.37-0.82] **	**-**	0.53 [0.34-0.85] *
Others		0.32 [0.07-1.44]		0.33 [0.07-1.51]
**Educational status**				
No-education	**-**	Ref	**-**	Ref
Primary	**-**	1.3 [0.91-1.80]	**-**	1.3 [0.90-1.78]
Secondary	**-**	2.3 [1.47-3.52] **	**-**	2.15 [ 1.38-3.34] *
Higher		3.2 [2.02-5.20] **	**-**	2.8 [1.73-4.53] **
**Wealth status**				
Poor	**-**	Ref	**-**	Ref
Middle	**-**	1.1 [0.72-1.77]	**-**	1 [0.66- 1.65]
Rich	**-**	2.1 [1.46-3.06] **	**-**	1.4 [0.90-2.09]
**Birth order**				
1^st^	**-**	Ref	**-**	Ref
2-3	**-**	0.66 [0.45-0.96]	**-**	0.65 [0.44- 0.94]
4-5	**-**	0.59 [0.33-1.07]	**-**	0.62 [0.34-1.13]
6+	**-**	0.39 [0.12-1.19]	**-**	0.39 [0.12-1.21]
**Parity**				
≤ 2	**-**	Ref	**-**	Ref
3-5	**-**	0.77 [0.51-1.17]	**-**	0.61 [0.52-1.22] *
2003≥ 6+	**-**	0.94 [0.33-2.66]	**-**	0.48 [0.36- 2.95]
**Contraceptive method**	**-**		**-**	
No-method	**-**	Ref	**-**	Ref
Traditional method	**-**	2.90 [1.08-7.82] *		2.6 [0.95-6.90]
Modern method	**-**	1.41 [1.08-1.84] *	**-**	1.4 [1.05-1.80] *
**ANC- visit**				
No-visit	**-**	Ref		
1-3 visit	**-**	2.11 [1.47-3.03] **		2.2 [1.51-3.12] **
4^th^ -visit	**-**	1.67 [1.13-2.47] *		1.7 [1.12-2.46] *
5+- visit	**-**	2.59 [1.80-3.74] **		2.4 [1.65-3.44] **
**Birth interval**				
<15	**-**	Ref	**-**	
15-30	**-**	1.14 [0.55-2.38]	**-**	1.2 [0.56-2.46]
>30	**-**	0.87 [0.42-1.78]	**-**	0.91 [0.44-1.85]
**Community- level variables**				
**Region**				
Tigray		-	**Ref**	Ref
Afar		-	0.46 [0.18-1.13]	1.9 [0.79-4.81]
Amhara		-	1.7 [0.77-3.74]	2.2 [ 1.07-4.45] *
Oromia		-	0.73 [0.31-1.65]	1.7 [0.80-3.87]
Somali		-	0.14 [0.04-.44] *	0.79 [0.24-2.58]
Benishangul		-	0.82 [0.33-1.98]	1.8 [0.81-4.13]
SNNPR		-	1.22 [0.55-2.72]	2.8 [ 1.26-6.00] *
Gambella		-	0.68 [0.27-1.69]	1.3 [0.52-3.00]
Harari		-	1.42 [0.62-3.23]	2.9[ 1.35-6.52] *
Addis Ababa			2.75 [1.19-6.34] *	3.4 [1.46-7.22] *
Dire dawa		-	2.32 [ 1.03-5.18] *	4.9[ 2.26-10.56] **
**Residence**				
Rural		-	Ref	Ref
Urban		-	3.7[ 2.36-5.63] *	1.6[ 1.04-2.57] *

*Key: Ref: Reference group; ***p***-value 0.05-0.01 *: ***p***-value < 0.01 *

NB: variable which were dropped during the consecutive tests were not included in the tables

## Discussion

The objective of this study was to assess the prevalence and associated factors of caesarian section in Ethiopia using the recent Ethiopian Mini Demographic and Health survey data set. The prevalence of caesarian section was 5.44% in the country in 2019. This finding is lower than that of studies conducted in Ghana 7% [34], Cameroon [35], Bangladesh [36], and lower recommended range of WHO which is 10-15%. It has been suggested that increasing CS up to 10% significantly reduces maternal and newborn deaths, but no change as proportion exceeds 10% [37].. From our finding (5.44%), it can be said that women in Ethiopia have either lower access or poorly afforded the service. It might mean a lot as life mothers and newborns might be losing because of the below required service magnitude. Inequality in accessing healthcare services might need to be the major agenda once again [7, 38].

This study revealed that as age of the women increase the likelihood of having caesarian section increased. This finding is supported by study done in Asia population [39], Bangladesh [40], Benin [41], and other countries [42]. This could be due to the fact that as women stay more in reproductive services, they might have good and experience of accessing CS and might also indicate that fetal growth impairment, pregnancy induced hypertension and maternal complications, and other obstetric and reproductive health problems increases with increased age [5, 22]. Having CS among Muslim and protestant women was less likely than that of orthodox women. This finding is in line with study in Nigeria [43]. This might indicate that service like CS might follow religious paths. .

Caesarian delivery was higher among women attended secondary and higher education than not educated women. This finding is supported with the similar studies in Ethiopian [20], Ghana [34], Benin [41]. The consistent information might indicate that educational status of the women remained the major factor in helping the women to make better self-reliant decisions.

Women who have three to five successful births were less likely to have caesarian section compared to the women who have one to two successful births. This is supported by some studies in the country [20, 44]. The possible explanation might be associated with the less experience of complications that lead to CS among women who gave spontaneous successful births previously. The odds of having CS among the women who use the modern contraceptive methods was higher compared to women of no contraceptive use [45]. This might be showing that mothers who used the modern contraceptive methods were the only mothers who have the knowledge, access, and can afford services. It might also be due to the fact that mothers who use modern contraception have long experience with healthcare services where they have been frequently for the specified service and that might made them a bit more knowledgeable than those who are not using. This might implied that increasing modern contraceptive utilization might trigger co-utilization of other services like CS [46, 47].

This study evidenced that those mothers who had ANC follow up were more likely to have cesarean section compared to mothers who have no- ANC visits. This finding is supported by the study done in Ghana [34]. It might be due to the exposure of ANC utilizing and exposure of mother to healthcare worker during ANC visits that helped them identify more risk of pregnancy complication [48, 49].

Women residing in Addis Ababa, Amhara, Dire Dawa, Harari and SNNP had higher odds of undergoing caesarian section compared to those residing in Tigray. This finding is supported by the previous study in Ethiopia [50]. This evidenced that the city administrators and agrarian regions have a good access to health facility services. This puts forth that there might be an inequality in accessing healthcare in some parts of the country. The women in urban area had higher odds of undergoing CS. This finding is supported by a study done in Nigeria [43]. the consistent information might be due to the urban women had access to skilled care providers, specialized hospitals, and obstetric services. In other words, this might be indicating that there was an inequality in accessing health services in the country. Although this study has vital information for the scientific community, it has also some limitations. The disproportionate sampling techniques and secondary source nature of the data were some of the limitations. We weighted the data and also collected all necessary documents to verify the appropriateness of the data before the analyses. Variables like respondent’s employment status, decision making, distance to health facility, and some other variables were not collected in the EMDHS 2019. In other words, we controlled for the effect of BMI to focus on access, availability, awareness, and affordability measuring factors. Thus, the result should be used in light of these limitations.

### Strength of the study

This study has a number of strengths. First, the study used recent data from a nationally representative demographic and health survey in Ethiopia, which allowed us to generalize the results to the rest of the country. We followed multilevel analysis to reduce intercommunity influences and data passed through various tests.

## Conclusion

Caesarian delivery in Ethiopia was not met the range of WHO recommendation. Women age, religion, educational status, parity, contraceptive method, ANC visit and community level factors region and place of residence were major determinants of caesarian delivery in Ethiopia. Empowering women, educating women, increasing co-services like modern contraceptive and ANC utilization and targeting mothers’ awareness might be very vital to deal with current problem. The prevalence of CS had regional variation the regional which also support the importance of region-specific further intervention.

## Supplementary Information

**Additional file 1.**

## Data Availability

The data in which the authors used to produce this manuscript are available upon reasonable request from the correspondence author

## Abbreviations

AOR: Adjusted Odds Ratio
CI: Confidence Interval
CS: Caesarian Section
EMDHS: Ethiopia Mini Demographic and Health Survey
ICC: Intra Class Correlation Coefficient
LLR: Log-Likelihood Ratio
MOR: Median odds ratio
OR: Odds Ratio
PVC: Proportional Change in Variance
SNNPR: Southern Nations, Nationalities, and Peoples’ Region
