# Supplementary material for: Prevalence and associated factors of caesarian section in Ethiopia: a multilevel analysis of the 2019 Ethiopia Mini Demographic Health Survey
Source: BMC Pregnancy Childbirth. 2021 Nov 30;21:798. doi: 10.1186/s12884-021-04266-7 (PMC8630861; doi:10.1186/s12884-021-04266-7)
Supplement: Supplementary file 1 — Additional file 1. [file 12884_2021_4266_MOESM1_ESM.doc]

STROBE Statement—Checklist of items that should be included in reports of ***cross-sectional studies***

|  | Item No | Recommendation |  |
| --- | --- | --- | --- |
| **Title and abstract** | 1 | Prevalence and associated factors of caesarian section in Ethiopia: a multilevel analysis of the 2019 Ethiopia Mini Demographic Health Survey | Page1 |
|  |  | Caesarian section is a vital emergency obstetric intervention for saving the lives of mothers and new borns. However, factors which are responsible for caesarian section (CS) were not well established in the country level data. Therefore, this study aimed to assess the prevalence and associated factors of caesarian section in Ethiopia. | Page2 |
| Introduction | | |  |
| Background/rationale | 2 | Caesarian section (CS) is the delivery of the pregnancy outcome by incision through the maternal abdomen and uterus which was named after the belief that Julius Caesar was born through CS. It has been recommended for the mothers whose labor progress is poor due to the either maternal or fetal factors | Page 4 |
| Objectives | 3 | Therefore, this study aimed to assess the prevalence and associated factors of caesarian section in Ethiopia. | Page 6 |
| Methods | | |  |
| Study design | 4 | Survey-based cross-sectional study design was employed | Page 7 |
| Setting | 5 | All nine regions and two city administrations of Ethiopia | Page 7 |
| Participants | 6 | All women (age 15–49) who give birth. |  |
| Variables | 7 | **Outcome measure:** caesarean delivery  Independent predictors were classified individual and community-level factors. Individual level factors were a religion, contraception, age, maternal education, household wealth index, place of delivery, parity, birth order, preceding birth interval, number of ANC visit and sex of the child. Community level factors were region and residence | Page 7 |
| Data sources/ measurement | 8* | DHS program (Demographic and Health Survey) website([www.measuredhsprogram.com](http://www.measuredhsprogram.com/)) after obtaining the necessary permissions for the download and further analyses. | Page 7 |
| Bias | 9 | Cross-sectional nature of the data prevents causality from being inferred between the independent and dependent variables |  |
| Study size | 10 | A total of 5527 study participant were involved in this study | Page 9 |
| Quantitative variables | 11 | Quantitative variables were handled in three level  Four models were constructed for multilevel logistic regression analysis using **melogit** STATA command**.** The first model (a random intercept model) was null model without predictors to determine the extent of cluster variations in anaemia. The second model (model I) was adjusted with individual-level variables. The third model (model II) was adjusted for community-level variables, while the fourth model (model III) was fitted with both individual-level and community-level variables simultaneously. | Page 8 |
| Statistical methods | 12 | Multilevel model for associated factors, we use mixed effect to control confounding than traditional regression model. | Page 8 |
|  |  | Spatial analysis was performed |  |
|  |  | For associated factor predictors with missing dropped and for spatial analysis without spatial information was dropped |  |
|  |  | Secondary data analysis and participants were selected based on a stratified two-stage cluster sampling technique |  |
|  |  | Spatiotemporal pattern analysis was done for three-year data |  |
| Results | | |  |
| Participants | 13* | A total of 5527 study participant were involved in this study | Page 9 |
| Descriptive data | 14* | A total of 5527 study participant were involved in this study. Out of this 300 (5.44%) were underwent caesarian section in the country in 2019. Among the eligible women for caesarian section 38 % were Muslim religion follower. More than half of the study participants were in age group of 15-29 (55.2 %) and had no education (53.58%). Majority of the women were from Oromia (40%), followed by SNNP (20%). Majority of the participants (95.77%)) were married and 45.56% of the women were poor | Page 9 |
|  |  | Husband educational level was missing data |  |
| Outcome data | 15* | The outcome variable was caesarean section delivery. It was retrieved from dichotomized DHS question asking for ‘delivery by caesarian section’. The responses were classified originally and here as No (0) or yes (1). | Page 7 |
| Main results | 16 | In the multilevel logistic regression analysis, women age, religion, educational status, parity, contraceptive method, ANC visit, region and place of residence were significantly associated with caesarian section delivery. The odds of CS among women in age group 30-39 and 40-49 years were 2.14 (AOR = 2.14, 95%CI = 1.55-2.94) and 2.20(AOR = 2.20, 95%CI = 1.20-3.97) times higher than that of the women in age group of 15-29 respectively. The odds of having CS among Muslim women (AOR = 0.50, 95%CI = 0.34-0.73) and Protestant (AOR = 0.53, 95%CI = 0.34-0.85) religion followers were decreased by 50% and 47 % compared to the Orthodox religion followers respectively. The odds of CS among women who educated secondary and higher level were 2.15 (AOR = 2.15, 95%CI = 1.38-3.34) and 2.8 (AOR = 2.8, 95%CI = 1.73-4.53) times higher than that of women with no education.  The odds of CS among mothers with 3-5 parity (AOR = 0.61, 95%CI = 0.44- 0.94) was decreased by 39% than that of mothers with 1-2 parity.  Women who use modern contraceptive methods had higher odds of CS (AOR = 1.4, 95%CI = 1.05-1.80) than women who had no contraceptive use experience. Women who used antenatal care of 1-3 visit, four visits, and five and above visits had higher odds of CS with AOR of (AOR = 2.2, 95% CI = 1.51-3.12), (AOR = 1.7, 95% CI = 1.12-2.46) (AOR = 2.4, 95% CI = 1.65-3.44) than those who do not have ANC respectively.  The odds of the CS for the women residing in Addis Ababa (AOR = 3.4, 95% CI = 1.46-7.22), Amhara (AOR = 2.2, 95% CI = 1.07-4.45), Dire Dawa (AOR = 4.9, 95% CI = 2.26-10.56), Harari (AOR = 2.9, 95% CI = 1.55-2.94), and SNNP (AOR = 2.8, 95%CI = 1.26-6.00) were higher than that of the women residing in Tigray. The odds of having CS among women of the urban resident was 1.6 (AOR = 1.6, 95% CI = 0.41, 0.45) relative to the rural resident | Page 10 |
|  |  | Not-applicable |  |
|  |  | Not-applicable |  |
| Other analyses | 17 | Descriptive and multi-level | Page 7 |
| Discussion | | |  |
| Key results | 18 | The ICC in the null model indicated that 41% of the total variability in caesarian section was attributed due to the differences between clusters while the remaining unexplained 59% of the total variability of caesarian section was attributed to the individual differences. Also, the MOR was 3.9 in null model which indicate that there was a variation in caesarian section between clusters. If we randomly select two women from different clusters, if we transfer women from low caesarian section clusters to higher caesarian section clusters, she might have 3.9 times higher odds of having caesarian section. This showed that the existence of significant heterogeneity in caesarian section delivery. The proportional change in variance (PCV) in this model was 77% which showed that 77% of community variance observed in the null model was explained by both community and individual level variables. The best fitted model was compared by deviance and the best fitted was model III, with the lowest deviance | Page 9 |
| Limitations | 19 | The cross-sectional nature of the data prevents causality from being inferred between the independent and dependent variables. | Page 13 |
| Interpretation | 20 | Women who have three to five successful births were less likely to have caesarian section compared to the women who have one to two successful births. This is supported by some studies in the country (20,44). The possible explanation might be associated with the less experience of complications that lead to CS among women who gave spontaneous successful births previously. The odds of having CS among the women who use the modern contraceptive methods was higher compared to women of no contraceptive use (45). This might be showing that mothers who used the modern contraceptive methods were the only mothers who have the knowledge, access, and can afford services. It might also be due to the fact that mothers who use modern contraception have long experience with healthcare services where they have been frequently for the specified service and that might made them a bit more knowledgeable than those who are not using. This might imply that increasing modern contraceptive utilization might trigger co-utilization of other services like CS (46)(47) | Page 13 |
| Generalisability | 21 | Caesarian delivery in Ethiopia was not met the range of WHO recommendation. Women age, religion, educational status, parity, contraceptive method, ANC visit and community level factors region and place of residence were major determinants of caesarian delivery in Ethiopia. Empowering women, educating women, increasing co-services like modern contraceptive and ANC utilization and targeting mothers’ awareness might be very vital to deal with current problem. The prevalence of CS had regional variation the regional which also support the importance of region-specific further intervention. | Page 13 |
| Other information | | |  |
| Funding | 22 | No funding |  |

*Give information separately for exposed and unexposed groups.

**Note:** An Explanation and Elaboration article discusses each checklist item and gives methodological background and published examples of transparent reporting. The STROBE checklist is best used in conjunction with this article (freely available on the Web sites of PLoS Medicine at http://www.plosmedicine.org/, Annals of Internal Medicine at http://www.annals.org/, and Epidemiology at http://www.epidem.com/). Information on the STROBE Initiative is available at www.strobe-statement.org.
